# Deciphering reproductive aging in women using a NOD/SCID mouse model for distinct physiological ovarian phenotypes

**DOI:** 10.18632/aging.205086

**Published:** 2023-10-16

**Authors:** María Marchante, Noelia Ramirez-Martin, Anna Buigues, Jessica Martinez, Nuria Pellicer, Antonio Pellicer, Sonia Herraiz

**Affiliations:** 1 IVIRMA Global Research Alliance, IVI Foundation, Valencia 46026, Spain; 2 Department of Pediatrics, Obstetrics and Gynecology, School of Medicine, University of Valencia, Valencia 46010, Spain; 3 Reproductive Medicine Research Group, Instituto Investigación Sanitaria La Fe (IIS La Fe), Valencia 46026, Spain; 4 IVIRMA Valencia, Valencia 46015, Spain; 5 IVIRMA Rome, Rome 00197, Italy

**Keywords:** age-related infertility, ovarian aging, mouse model, oocyte quality, embryo development, mitochondrial function

## Abstract

Female fertility is negatively correlated with age, with noticeable declines in oocyte quantity and quality until menopause. To understand this physiological process and evaluate human approaches for treating age-related infertility, preclinical studies in appropriate animal models are needed. Thus, we aimed to characterize an immunodeficient physiological aging mouse model displaying ovarian characteristics of different stages during women's reproductive life. NOD/SCID mice of different ages (8-, 28-, and 36–40-week-old) were employed to mimic ovarian phenotypes of young, Advanced Maternal Age (AMA), and old women (~18–20-, ~36–38-, and >45-years-old, respectively). Mice were stimulated, mated, and sacrificed to recover oocytes and embryos. Then, ovarian reserve, follicular growth, ovarian stroma, mitochondrial dysfunction, and proteomic profiles were assessed. Age-matched C57BL/6 mice were employed to cross-validate the reproductive outcomes.

The quantity and quality of oocytes were decreased in AMA and Old mice. These age-related effects associated spindle and chromosome abnormalities, along with decreased developmental competence to blastocyst stage. Old mice had less follicles, impaired follicle activation and growth, an ovarian stroma inconducive to growth, and increased mitochondrial dysfunctions. Proteomic analysis corroborated these histological findings. Based on that, NOD/SCID mice can be used to model different ovarian aging phenotypes and potentially test human anti-aging treatments.

## INTRODUCTION

Given the increased life expectancy of advanced society, deciphering the multifaceted aging process can help prevent or reverse age-associated phenotypes of several tissues and/or organs [1, 2]. Unveiling the molecular mechanisms that underly aging is of utmost importance for women of advanced maternal age (AMA), as their ovaries undergo an accelerated aging process [3]. Indeed, the significant decline in ovarian reserves after 35 years of age compromises the quantity and quality of oocytes [4], and ultimately, the ability to achieve pregnancy with autologous oocytes [5].

The complexities of the causal mechanisms of ovarian aging remain unknown. However, DNA damage, mitochondrial dysfunction, errors in meiotic recombination and spindle assembly, and senescence, among others, have been proposed to be responsible for age-related ovarian changes [6–9]. The development of suitable animal models can help elucidate the etiology of aging and validate new strategies that delay or reverse the effects of human ovarian aging [10]. Among the different models employed to study aging, *Mus musculus* is the most widely used, due to its homology to humans [11] and reproductive characteristics [12].

Mouse models established by gene deletion, chemotherapy or radiation have been used for studying ovarian aging [13]. Genetic engineering grants us the ability to study the effects of specific gene alterations on ovarian aging. In this regard, distinguished knockout models include those with the BRCA2 receptor deficiency [14], follicle-stimulating hormone receptor depletion [15], loss of pigment epithelium-derived factor [16], or mitochondrial genes deletion [17–19] employed to decipher potential mechanisms of ovarian aging. However, these models present some limitations. Indeed, most of them display an impairment of ovarian reserve and folliculogenesis from the early stages of development and therefore, do not mimic reproductive systems in humans. In addition, these models are expensive and complex to generate, thus the use of other animal models such as those induced by chemotherapy (ChT) is still required. Cyclophosphamide, busulfan, and paclitaxel are commonly used to establish murine models of ovarian damage [20], which facilitate the study of the gonadotoxic effects of chemotherapy on fertility outcomes, like follicle loss and oocyte death, and are crucial in the context of fertility preservation, to design ovoprotective strategies for cancer patients [21–23]. Clinically, the effect of cytotoxic treatments on the ovary can range from partial damage that reduces fertility, to the destruction of the follicular pool and tissue atrophy, leading to premature ovarian insufficiency (POI) and a loss of fertility [24]. The degree of gonadotoxicity differs depending on the ChT drugs and dose, with alkylating drugs carrying the highest risk of ovarian failure [25]. In addition, accelerated ovarian aging can be induced by radiation [26, 27] or by a combination of chemotherapy and radiation [28].

Although knockout and chemotherapy-induced models enable the study of characteristics derived from aging and POI conditions, they do not represent a progressive and physiological aging mouse model. Thus, we aimed to establish and characterize an immunodeficient murine model for physiological reproductive aging, that manifests the ovarian phenotypes corresponding to the different stages of women’s reproductive life. Among the different mouse strains employed in preclinical studies, the use of immunodeficient mice [29] such as the NOD/SCID strain, will allow us to evaluate potential therapies of human origin to slow down or reverse the physiological ovarian aging process [30–34].

## RESULTS

### Older mice present a decreased ovarian reserves, follicular activation and growth, and deteriorated ovarian stroma

NOD/SCID female mice of 8 (young group), 28 (AMA group), and 36 weeks (old group) of age were employed to mimic the ovarian phenotypes of young (~18–20 years old), AMA (~36–38 years old), and reproductively-aged (>45 years old) women, respectively.

**Figure 1:**
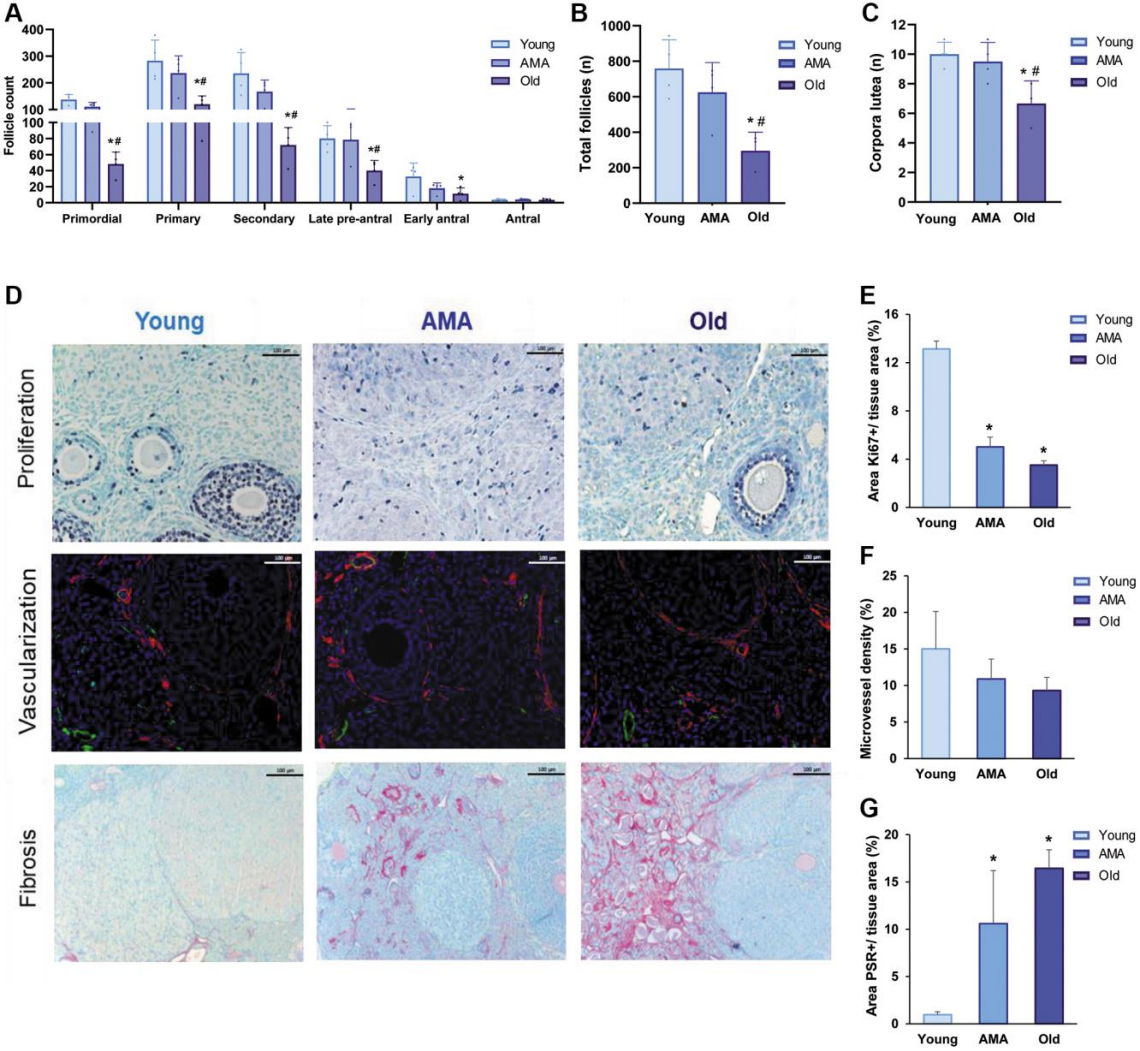
**Histological analysis of the follicle reserve and ovarian stroma of a NOD/SCID mouse model for physiological human aging.** (**A**) The follicular analysis of primordial, primary, secondary, late pre-antral, early antral, and antral follicles in young, advanced maternal age (AMA), and old mice notices a negative impact of age. The total number of follicles (**B**) and corpora lutea (**C**) are also affected. (**D**) Photomicrographs of the ovarian stroma, showing Ki67-positive proliferative cells in purple (top row), isolectin-B4-positive endothelial cells in green and a-smooth muscle actin in red (middle row), and collagen fibrils in red (bottom row). The black and white scale bars are set to 100 μm. Quantification of proliferation (**E**), vascularization (**F**), and fibrosis (**G**) in the ovaries of young, AMA, and old mice shows that the effects of age are mirrored in the ovarian stroma. All analyses were based on four samples per group. ^*^p < 0.05 AMA and old vs. young, ^#^*p* < 0.05 Old vs. AMA.

Our model mimicked depletion of the ovarian reserve with advanced age. We observed significant reductions in the number of primordial, primary, and growing follicles in old mice, compared to young and AMA groups (*p* < 0.05; Figure 1A), which mirrored the decrease in the total number of follicles (young vs. old, *p* = 0.0078; AMA vs. old, *p* = 0.03; Figure 1B). Accordingly, we found less corpora lutea in the ovaries of old mice (young vs. old, *p* = 0.013; AMA vs. old, *p* = 0.044; Figure 1C). These phenomena were supported by a reduced activation of primordial follicles, evidenced by the significant increase of FOXO3 nuclear localization, in both the AMA and old mice, compared to the young controls (43 ± 3% and 22 ± 7% vs. 53 ± 5%; *p* = 0.049 and *p* = 0.003, respectively).

Age-related deficiencies were also noted in the ovarian stroma, with significantly lower cell proliferation in the ovaries of AMA (*p* < 0.01) and old mice (*p* < 0.01), with respect to young mice (Figure 1D, 1E). Particularly, the percentage of proliferative primary (25% and 28% vs. 47%, respectively) and secondary follicles (71% and 37% vs. 80%, respectively) gradually decreased with age, corresponding with the trend for reduced microvessel density in older mice (*p* > 0.05; Figure 1D–1F), and significantly augmented fibrosis in AMA (*p* = 0.04) and old mice (*p* = 0.01), compared to the young mice (11 ± 6% and 16 ± 2% vs. 1 ± 0.3%, respectively; Figure 1D–1G).

### Age affects reproductive potential, decreasing the quantity and quality of harvested oocytes and embryos

**Figure 2:**
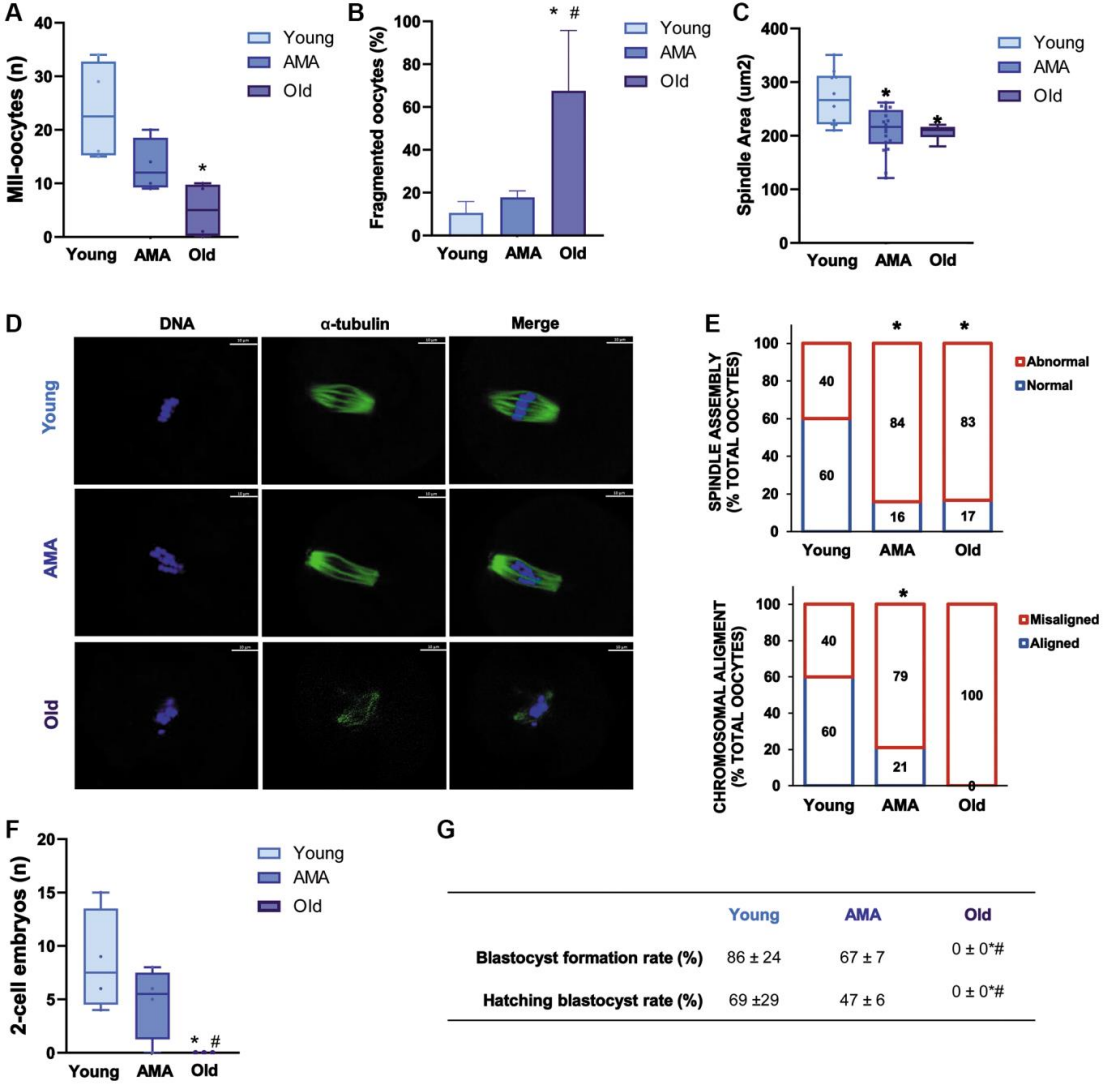
**Reproductive outcomes of a NOD/SCID mouse model for physiological human aging.** The number of metaphase-II (MII) oocytes (**A**) and percentage of fragmented oocytes (**B**) recovered from young, advanced maternal age (AMA), and old mice, following controlled ovarian stimulation (COS) are decreased by age. The oocyte quality is also affected by age reducing (**C**) spindle area in AMA oocytes. (**D**) Representative immunofluorescence images of oocyte quality analysis, showing alpha-tubulin (green) and chromosomes (blue). The white scale bars are set to 10 μm. (**E**) The proportion of oocytes with normal vs. abnormal spindle assembly (top) and aligned vs. misaligned chromosomes (bottom) are modified with age. At the time of collection, the number of recovered 2-cell embryos (**F**) is lower in aged mice. (**G**) Blastocyst formation and hatching rates following *in vitro* embryo culture are also impaired. All analyses were based on four samples per group. ^*^p < 0.05 AMA and Old vs. Young; ^#^*p* < 0.05 Old vs. AMA.

Following COS, less MII oocytes were recovered from the mice of AMA than young mice (13 ± 5 vs. 24 ± 9, respectively; *p* > 0.05). Notably, this trend was significantly enhanced with the old mice (5 ± 5 vs. 24 ± 9, respectively; *p* = 0.014, Figure 2A) who had 67.5% of oocytes with fragmented intracellular contents as a consequence of reduced quality (Figure 2B). In-depth immunofluorescence-based assessment of oocyte quality showed significantly decreased spindle area (Figure 2C, 2D) and abnormal spindle assembly (Figure 2E) in both the oocytes of AMA and old mice compared to the young group (*p* < 0.05). Accordingly, we found a higher proportion of oocytes with chromosomal misalignments in AMA and old groups (79% and 100% vs. 40%, respectively; Figure 2E).

In a similar manner, fewer 2-cell embryos were harvested from mice of AMA compared to young mice, but the difference was not statistically significant (5 ± 3 vs. 9 ± 5, respectively; Figure 2F). Subsequent *in vitro* embryo culture revealed that blastocyst formation and hatching were respectively impaired by 19% and 22% in the AMA mice compared to the young mice (Figure 2G). Notably, no viable embryos were recovered from old mice, substantiating the significant implications of age on reproductive function (Figure 2F, 2G).

### The progressive ovarian aging is associated with a reduced number of mitochondrial copies and increased oxidative damage and apoptosis

**Figure 3:**
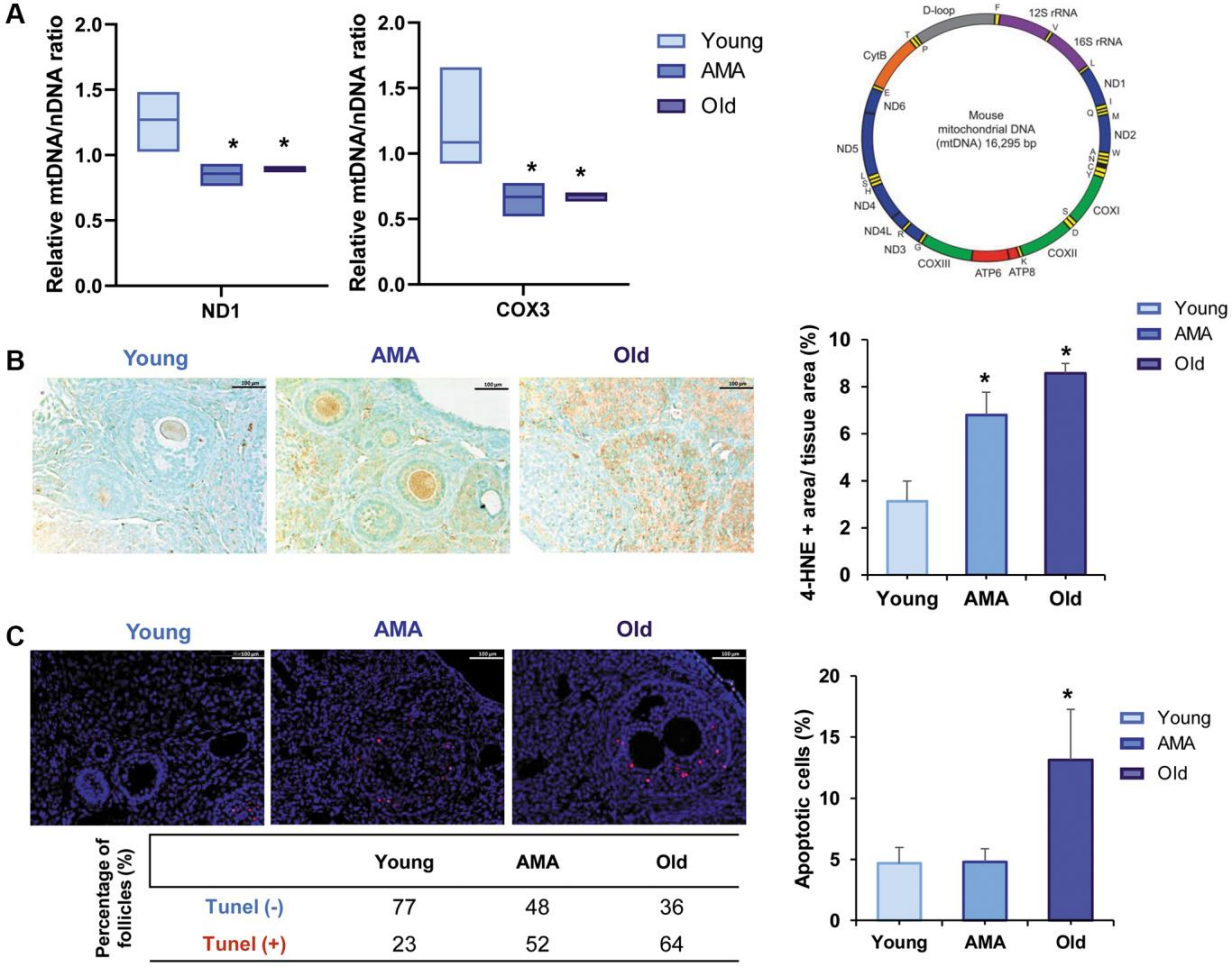
**Mitochondrial function in the ovaries of a NOD/SCID mouse model for physiological human aging.** (**A**) Relative mitochondrial/nuclear DNA (mtDNA/nDNA) ratio in the ovarian tissue of young, advanced maternal age (AMA) and old mice, based on the RT-qPCR amplification of *ND1* and *COX3* mitochondrial genes normalized to the nuclear *18S* gene shows a reduced number of mitochondrial copies in aged groups. Note, *ND1* and *COX3* were selected from the stable regions of the mouse mtDNA (depicted as the purple, blue, and green segments). (**B**) Photomicrograph of ovarian sections shows oxidative damage (brown) visualized with the immunohistochemical staining of the peroxidative lipid product 4-hydroxynonenal (4-HNE), and corresponding quantification of the damaged tissue is higher in older mice. The black scale bars are set to 100 μm. (**C**) Representative immunofluorescent images of cell death (indicated by TUNEL-positive red signal), and the corresponding quantification of apoptotic cells notice more apoptosis in older mice. White scale bars are set to 100 μm. All analyses were based on four samples per group. ^*^p < 0.05 AMA and Old vs. young, ^#^*p* < 0.05 Old vs. AMA.

Based on the relative expression of *ND1* and *COX3* mitochondrial genes, we estimated there were significantly less mtDNA copies in AMA and old ovaries compared to the young group (*p* < 0.05; Figure 3A). In the old mice, fewer mitochondrial copies were also associated with a significant overexpression of 4-HNE, a marker of lipid peroxidation and oxidative damage (Figure 3B), and abundance of apoptotic cells (*p* = 0.028) and follicles compared to young mice (64% vs. 23%, respectively; Figure 3C).

### Age-related changes are supported by the ovarian proteomic profile

**Figure 4:**
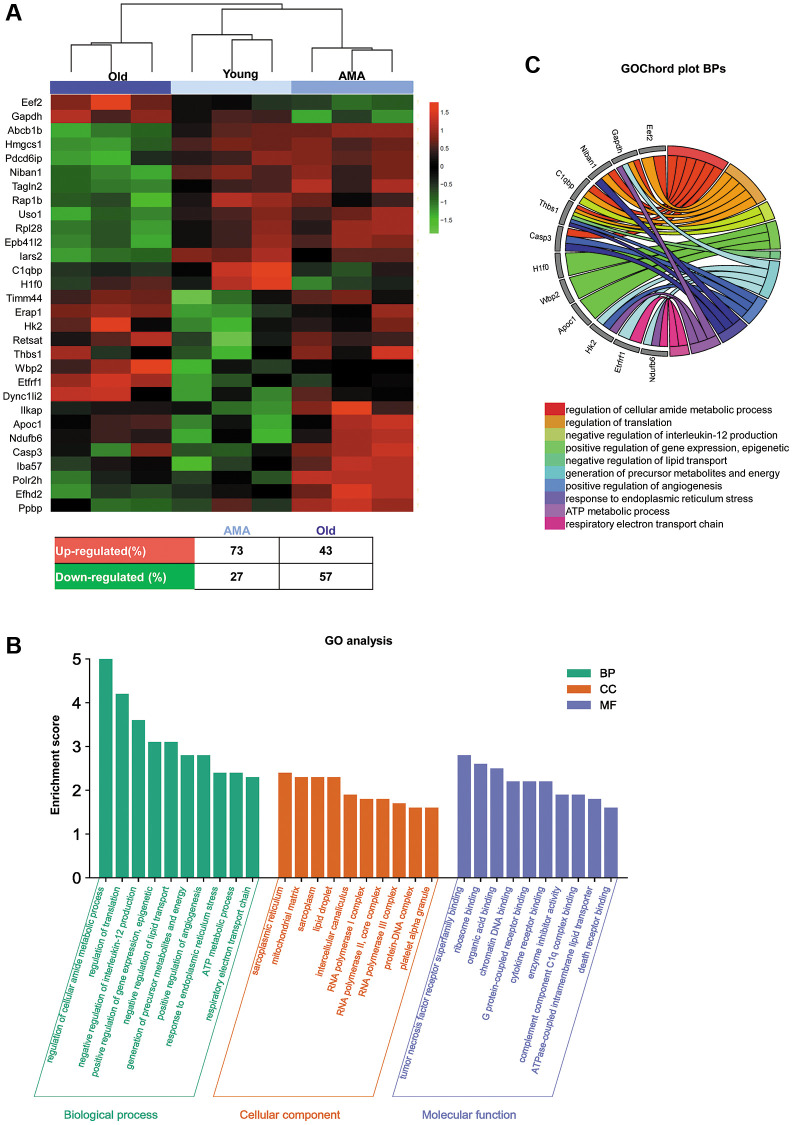
**Ovarian proteomic profiling of a NOD/SCID mouse model for physiological human aging.** (**A**) Hierarchical clustering based on the mean expression of the 30 significantly differentially expressed proteins (DEPs) between young, advanced maternal age (AMA), and old mice identified by ElasticNET regression analysis. The table beneath the heatmap shows an overview of the DEPs from the AMA and old mice, with respect to the young mice. (**B**) Significantly enriched Gene Ontology (GO) biological processes, cellular components, and molecular function (FDR <0.05) associated with the DEPs identified in each group. (**C**) GOChord plot of the top ten biological processes for young, AMA, and old mice, showing the relationship between pathways and the most relevant genes between them.

SWATH analysis was used to study the age-related modifications in the ovarian proteome. A total of 1,834 proteins were quantified among the ovaries of young, AMA, and old mice (Supplementary Table 1). ElasticNET regression analysis identified 30 DEPs, which broadly clustered by age (i.e., old vs. young and AMA groups), and distinguished between young and AMA sub-clusters (Figure 4A). Remarkably, we observed a global downregulation of proteins in the old and AMA mice, with respect to the young mice (57% and 27%, respectively).

Next, we studied the functionality of the DEPs by performing a GO analysis (Figure 4B, 4C). The GO enrichment revealed biological processes related to the regulation of metabolic processes, gene expression, lipid transport, and angiogenesis, in addition to pathways involved in mitochondrial activity, such as response to stress, ATP metabolic processes, and respiratory electron transport chain. Enriched cellular components included the sarcoplasmic reticulum, mitochondrial matrix, intercellular transport, polymerase complexes, and DNA binding proteins. Among the enriched molecular functions, we found the tumor necrosis factor receptor superfamily, chromatin DNA, death receptor, and complement component C1q complex. Finally, GOChord plot analysis highlighted *Casp3*, *C1qbp*, *Thbs1*, and *Gapdh* genes among the top ten enriched biological processes (Figure 4C).

### Cross-validation of age-related effects on oocyte retrieval and early embryo development after COS in C57BL6 mice

C57BL6 mice were employed to cross-validate the main age-related effects on fertility outcomes after COS observed in the NOD/SCID strain. The number of harvested oocytes and embryos and subsequent embryo culture to the blastocyst stage were assessed.

**Figure 5:**
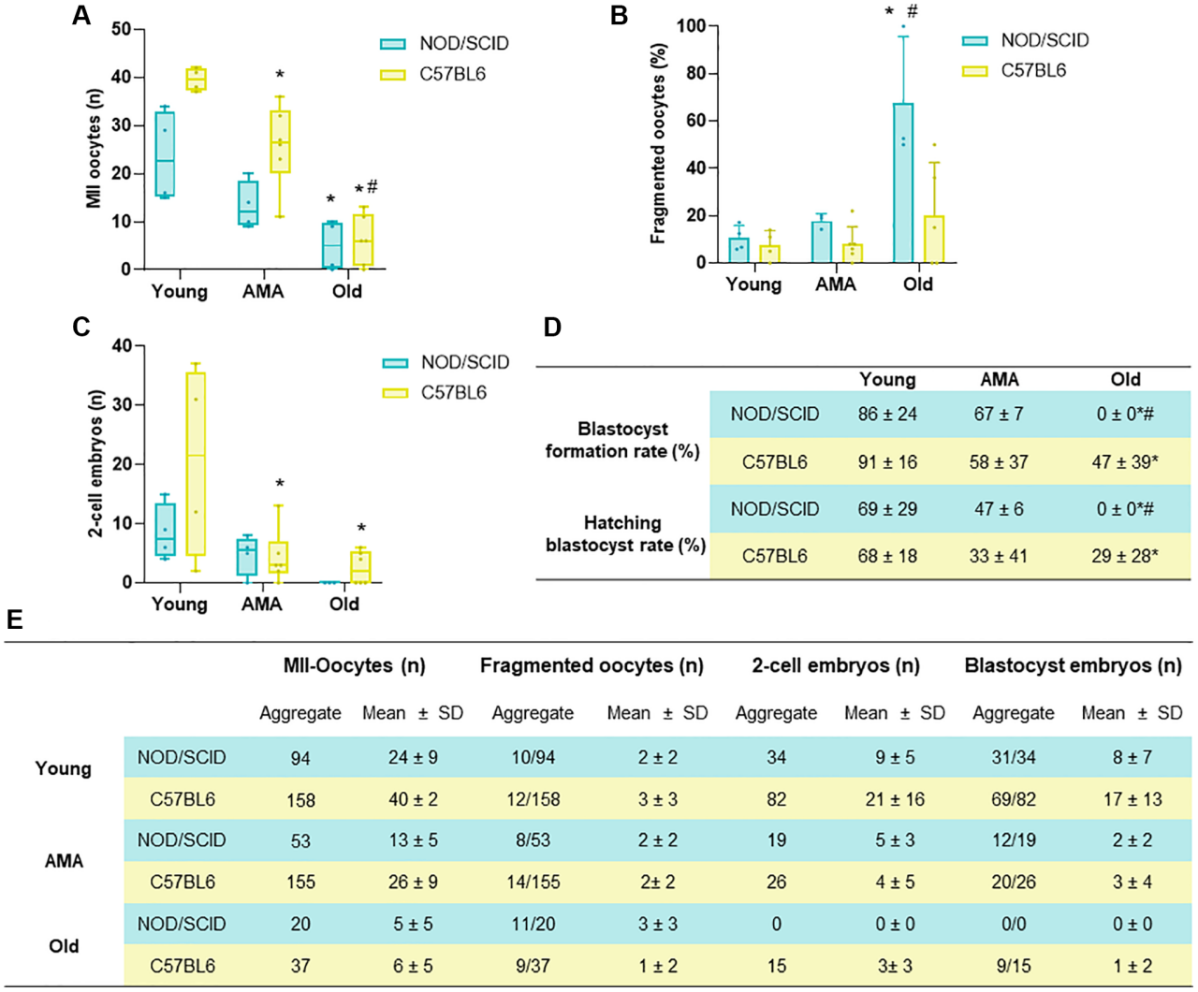
**Comparison of the reproductive outcomes of NOD/SCID and C57BL/6 mice.** (**A**) The number of metaphase (MII)-oocytes, (**B**) fragmented oocytes, and (**C**) 2-cell embryos recovered following controlled ovarian stimulation (COS) in young, Advance maternal age (AMA), and old mice from NOD/SCID and C57BL/6 showed similarities between strains. (**D**) Blastocyst formation and hatching rates after *in vitro* embryo culture were affected by age in both mice strains. (**E**) Summary table presenting aggregate data and mean+ SD per mouse for the number of MII-oocytes, fragmented oocytes, 2-cell embryos and blastocysts in both strains tested. *N* = 4–6 mice per group. ^*^p < 0.05 AMA and Old vs. Young, ^#^*p* < 0.05 Old vs. AMA.

A reduced number of MII oocytes was collected from the AMA mice compared to the young group (40 ± 2 vs. 26 ± 9, respectively; *p* = 0.016), being this effect enhanced in the old mice, where MII yield was lower than in AMA and young mice (6 ± 5, *p* < 0.001 and *p* < 0.0001, respectively; Figure 5A). Moreover, a higher percentage of the ovulated oocytes had poor quality with fragmented intracellular contents in the old group (7% and 8% vs. 20%, *p* = NS; Figure 5B).

These deleterious effects were also reflected in the number of 2-cell embryos recovered from oviducts in AMA and Old groups compared to young mice (21 ± 16 vs. 4 ± 5 and 3 ± 3, *p* < 0.05; Figure 5C). Indeed, further embryo *in vitro* culture showed an impaired blastocyst formation and hatching rates in AMA (58% and 33%) and especially in old (47% and 29%) groups compared to the young (91% and 68%, *p* < 0.05; Figure 5D, 5E).

## DISCUSSION

This study characterized the reproductive outcomes of young, AMA and old NOD/SCID mice modeling the physiological ovarian aging of humans. The main purpose of our study was to establish a physiological ovarian aging mouse model that could be employed to evaluate potential therapeutic interventions derived from human origin. Therefore, the use of an immunodeficient mouse strain, such as the NOD/SCID, was essential. Older mice of this strain presented with reduced ovarian reserves, follicular activation, and growth, deteriorated ovarian stroma, and decreased quantity and quality of harvested oocytes and embryos following COS. Based on histological analyses, we established associations between progressive ovarian aging and a reduced number of mitochondrial copies, oxidative damage, and apoptosis, and these age-related changes were corroborated at the proteomic level.

Our model of physiological and progressive ovarian aging transcends previous models of ovarian disorders established by chemotherapeutic agents or gene deletions, which compromise the ovarian reserve and inaccurately represent the genetic conditions of aging [15–17, 21–23, 35, 36]. The mice mimicked the ovarian phenotypes of young, AMA, and reproductively-aged women, allowing us to assess the histological and proteomic changes associated with physiological ovarian aging, and begin to reveal the underlying mechanisms. Due to its immunodeficient nature, our model has the potential to become a valuable preclinical tool to test a broad variety of anti-aging treatments of human origin, including human blood-based therapies such as stem cells or plasma [37–40], which may be used to treat the growing number of patients with age-related ovarian infertility.

Comparing the ovarian reserve across the three groups, we found a significantly reduced number of primordial and primary follicles in old mice. Moreover, the analysis of activated and growing follicular populations revealed an impairment of folliculogenesis in aged ones, respectively corresponding with the decline in ovarian reserve and reduced efficiency of COS reported for older women [41]. Accordingly, the age-related effects were also evident in the ovarian stroma of AMA and old mice, with enhanced fibrosis, and reduced proliferation and vascularization. These findings agreed with previous reports of increased fibrosis [42] and cellular senescence [43, 44] in aged mammalian ovaries, which limit cellular proliferation and follicle growth. Moreover, ovaries from older individuals have fewer blood vessels, hindering the delivery of oxygen and nutrients to growing follicles [45, 46]. Altogether, these results establish a link between the age-associated deficiencies of the ovarian stroma, follicular development, and, ultimately, ovarian function.

Following COS, we recovered fewer oocytes, of poorer quality, from older mice. This poor oocyte quality was also reflected by the reduced number, or absence, of 2-cell embryos recovered in the AMA and old groups, respectively. As observed in patients over the age of 35 who experience age-related fertility problems, the limited quantity and compromised quality of oocytes is significant [5], and leads to a marked reduction in fertilization and pregnancy rates [47, 48]. Accordingly, we found embryo development to the blastocyst stage was impaired in the AMA and old groups. Moreover, in our study we observed increased abnormal spindle assembly and chromosomal misalignments in AMA and old groups. Spindle and chromosomal abnormalities in oocytes are more prevalent with maternal aging, increasing the risk of aneuploidy and being used as feasible marker of oocyte quality [49, 50]. Thus, the lack of viable embryos obtained is likely due to poor oocyte quality [51], oocyte spindle dysfunction and meiotic errors [52, 53], that have previously been described among aged women.

Mitochondrial dysfunction is a principal contributor to the aging process [6, 54], via overproduction of reactive oxygen species (ROS) that damage the ovarian tissue. Notably, lipid peroxidation, and its product 4-HNE, are responsible for the oxidative damage that disrupts folliculogenesis and oocyte meiosis [55, 56] and contributes to the accumulation of spindle assembly defects and chromosome misalignments [57]. On the other hand, the mtDNA copy number is a promising biomarker, that has previously been correlated with lower oocyte quality [58–61]. There were significantly reduced mitochondrial copy numbers and significantly increased lipid peroxidation and apoptosis in the ovaries of AMA and old mice, with respect to young mice, supporting previous reports of age-related decreases of mtDNA levels, increased ROS, oxidative damage, and apoptosis, that altogether have adverse effects on oocyte quality [56, 62].

Remarkably, our histological findings were strongly supported by proteomic analysis, showing distinct expression profiles in the ovaries of young, AMA, and old mice. Functional analysis of the DEPs revealed significant alterations in various processes as mice age. Particularly, these changes were associated with the stress response, respiratory electron transport chain, the death receptor binding, complement component 1q binding, and chromatin DNA binding. Indeed, increased in stress response molecules has been previously linked to aging, representing a compensatory mechanism to counterbalance heightened stress and maintain cellular and organismal homeostasis [63, 64]. Furthermore, a defective electron transport chain emerged as a pivotal mechanism in ovarian aging by reducing the mitochondrial energy metabolism, diminishing bioenergetics capacity and increasing oxidative stress [7]. Multiple studies also underscore the relevance of DNA repair and replication genes in the age of natural menopause and ovarian aging [65, 66]. In addition, older mice exhibited an overexpression of pro-apoptotic and age-related cell death genes, such as *Casp3*, while repressing those genes associated with damage prevention, vascularization, and cell survival, such as *C1qbp* [67], *Thbs1* [68], and *Gapdh* [69], respectively. Therefore, our proposed NOD/SCID mouse model effectively recapitulates many of these previously established alterations associated with ovarian aging, highlighting the validity of our NOD/SCID mouse model as a valuable tool for future investigations in this field.

Finally, to cross-validate our findings and corroborate the suitability of the established age groups and the response to ovarian stimulation, we assessed the reproductive outcomes of age-matched C57BL/6 mice, which are also commonly used in preclinical reproductive medicine studies. Similar to the NOD/SCID strain, the C57BL/6 mice of AMA and old groups also showed a reduced quantity of MII oocytes and embryos after COS along with an impaired *in vitro* embryo development until the blastocyst stage, suggesting that the findings from our immunodeficient ovarian aging mouse model match with other mouse strains. However, further characterization of the ovarian microenvironment (e.g., quality of the stroma, vasculature, and cell death) would be necessary to corroborate the effect of ovarian aging observed in this C57BL/6 strain, or others. Interestingly, the impact of age on the reproductive outcomes in C57BL/6 females was not as pronounced as in the NOD/SCID strain, suggesting an accelerated aging process in these immunodeficient mice. Previous studies showed that alterations in the immune response are among the major contributors of ovarian aging [70], demonstrating a strong relationship between an impairment of immune system decline and the age-related ovarian function decline [70–72]. Thus, the accelerated aging observed in NOD/SCID mice, compared to the C57BL/6 strain, can be attributed to their immunodeficient status.

In summary, in this study we characterized the quality of the ovarian microenvironment and reproductive outcomes of an immunodeficient murine model of physiological ovarian aging by evaluating fertility outcomes, ovarian reserve and stroma, mitochondrial dysfunctions, and the ovarian proteome at different stages. This model adequately mimicked the characteristics of the reproductive stages in women, without external agents compromising folliculogenesis, or disrupting molecular mechanisms and ovarian function, which could mask the processes of physiological aging. Nevertheless, additional studies elucidating the molecular mechanisms underlying the age-related changes in signaling pathways and biological processes could help unravel the complexities of this multifactorial physiological process. Our study confirms that immunodeficient NOD/SCID mice of established ages can be employed to model the physiological aging of human ovaries. Indeed, our murine model mimicked the ovarian phenotypes of young, AMA, and old women, highlighting the histological and proteomic characteristics of each stage.

As such, we put forth this commonly used laboratory mouse strain as a preclinical model that may feasibly be employed to further decipher the complexities of ovarian aging, or test novel and alternative anti-aging treatments to prolong women’s fertility.

The proposed NOD/SCID model, with its accelerated ovarian aging, holds particular value as it will enable the observation of ovarian changes within a shorter timeframe. This characteristic is highly beneficial for future studies as it will reduce the time required to obtain reproductively aged mice, thus optimizing research efficiency.

## MATERIALS AND METHODS

### A mouse model for ovarian aging

Twelve female NOD/SCID mice (Janvier-Lab, Le Genest-Saint-Isle, France) of 8 (*n* = 4), 28 (*n* = 4), and 36 weeks old (*n* = 4), were employed to mimic the ovarian phenotypes of young (~18–20 years old), AMA (~36–38 years old), and old (>45 years old) women, respectively. The different ages were established considering the characteristics of the NOD/SCID strain and the accelerated aging that animals suffer because of their immunodeficient condition [73], and the approximate mouse/human age comparisons established by Fluerker et al. 2007 [10, 73], in which the authors consider: birth-1 month, mice develop 150 times faster than humans, 1–6 months, mice mature 45 times faster; >6 months, mice age 25 times faster.

Once the animals reached the target age, controlled ovarian stimulation (COS) was initiated with 10 IU of pregnant mare serum gonadotropin (PMSG, Sigma-Aldrich, St. Louis, MO, USA), followed by a 10 IU injection of human chorionic gonadotropin (hCG, Sigma-Aldrich, St. Louis, MO, USA) 48 h later. Then, animals were mated (2 females:1 male) and sacrificed by cervical dislocation 36 h after hCG injection to recover the ovulated metaphase-II (MII) oocytes and 2-cell embryos directly from the oviduct. The ovaries were harvested to compare the follicular growth, quality of the ovarian stroma, mitochondrial function, and proteomic profiles between the different ages, as detailed in the following sections (Supplementary Figure 1). One ovary was immediately fixed for subsequent histological analysis and immunostaining, while the other was snap frozen at −80ºC for molecular analysis. During the experiment, all mice were maintained with *ad libitum* access to a standard diet, and housed in a specific pathogen-free zone in a 12:12 h light:dark cycle.

The age-related effects on oocyte retrieval and early embryo development were also evaluated in 8-, 28- and 36-week-old C57BL/6 mice (*n* = 16), to cross-validate the reproductive outcomes observed in our physiological aging model.

### Characterization of ovarian reserves and folliculogenesis

The formalin-fixed ovaries were embedded in paraffin, serially sectioned into 4-μm slices, and stained with hematoxylin and eosin (H&E) to count follicles in every fifth section. Follicle subpopulations (i.e., primordial, primary, secondary, late preantral, early antral, and antral follicles) and corpora lutea were quantified, according to established morphological criteria [30, 74]. The localization of FOXO3, a marker of oocyte activation, was evaluated with immunostaining; primordial follicles were considered activated when FOXO3a was located in the ooplasm [75].

### Analysis of the ovarian stroma: proliferation, vascularization, and fibrosis

Ki-67 immunostaining was used to examine proliferative stromal and follicular cells [32], while the microvessel density was assessed by double immunofluorescence against isolectin B4 and smooth muscle actin, as previously reported [30]. Collagen deposits were analyzed using the Picrosirius Red Stain Kit (PSR, Polysciences, Warrington, PA, USA), according to the manufacturer´s instructions [76]. Multiplex high-magnification images (20X) were captured from four ovarian sections of each mouse, using an optical microscope with a digital camera (LEICA DM4000B and DFC450C; Leica Microsystems GmbH, Germany), and quantified using Image-Pro Plus software (Media Cybernetics, Carlsbad, CA, USA). In each section, cell proliferation was estimated by dividing the Ki-67 positive area by the area of ovarian tissue, while ovarian vascularization was estimated by dividing the lectin-positive area by the area of ovarian tissue, indicating new microvessel formation. Ovarian fibrosis was calculated using the PRS-positive signal normalized to the area of ovarian tissue per section.

### Oocyte and embryo collection

After sacrifice, the oocytes and embryos were flushed from the oviduct using a 30 G needle and global collect medium (GCOL-100, CooperSurgical Fertility and Genomic Solutions, Denmark). Under a binocular loupe, the collected oocytes and embryos were evaluated, quantified, and classified, as described in the following sections. Oocytes with a polar body extrusion were considered mature, while those with fragmented ooplasm were classified as bad-quality oocytes.

#### Oocyte quality assessment

MII oocytes were fixed in a supplemented PHEM buffer (Supplementary Table 2) for 20 minutes, washed and incubated overnight in blocking solution (Supplementary Table 2). The spindle microtubules and chromosomes were respectively labeled with an anti-tubulin FITC antibody (1:50; Sigma-Aldrich, St. Louis, MO, USA) and Hoechst 33342 (20 g/mL; Sigma-Aldrich). Oocytes were then placed in drops of phosphate-buffered saline, under mineral oil, in glass-bottom dishes (Ibidi, Germany). Serial Z-section images were taken with a confocal microscope (LEICA TCS-SP8), using an oil immersion objective lens (40X–60X). Spindle area and chromosome alignment were evaluated using ImageJ software [77]. Oocytes were considered normal when they presented chromosomes aligned on the metaphase plate and well-organized microtubules, or abnormal when they had disordered spindles and/or unaligned chromosomes.

#### In vitro embryo development

The 2-cell embryos were cultured in GPS dishware with Sage 1-step media (both from CooperSurgical Fertility and Genomic Solutions, Denmark) at 37°C with 5% O_2_, 6% CO_2_, and 89% N_2_. Embryos were evaluated on day 5–6 of *in vitro* culture, to assess blastocyst formation and hatching.

### Assessment of mitochondrial function

#### Mitochondrial DNA (mtDNA) copy number

Mitochondrial DNA (mtDNA) copies in the ovarian samples were estimated using a real-time quantitative polymerase chain reaction (RT-qPCR)-based method. Briefly, total DNA was extracted from half frozen ovaries, using the QIAamp kit (Qiagen, Germantown, MD, USA), according to manufacturer’s instructions. Specific primers were designed to target two mitochondrially-encoded genes, *ND1* and *COX3* (Supplementary Table 3), and the nuclear small subunit 18S rRNA (*18S*). RT-qPCRs were performed using PowerUp SYBR Green on a StepOnePlus System (Applied Biosystems, Foster City, CA, USA), with the optimized cycling parameters detailed in Supplementary Table 3. The mtDNA/nuclear DNA (nDNA) ratio was calculated using the ΔΔCt method.

#### Oxidative stress and apoptosis

Immunohistochemical staining of the lipid peroxidation product 4-hydroxy-2-noneal (4-HNE) was performed on ovarian sections, to localize oxidative damage. Briefly, samples were incubated with anti-4-HNE antibody overnight at 4°C (1:500; Abcam, Cambridge, UK), followed by goat anti-rabbit secondary antibody conjugated to horseradish peroxidase (HRP) enzyme (1:1000; Vector Laboratories, Burlingame, CA, USA) for 1 h at RT. Positive staining was detected using a 3,3’-diaminobenzidine (DAB) peroxidase substrate kit (Dako Denmark A/S, Glostrup, Denmark). Human kidney sections were used as negative controls. Ovarian sections were imaged using bright-field microscopy and the expression of 4-HNE was quantified with Image-Pro Plus. The oxidative damage was calculated by dividing the 4-HNE positive area by the total area of ovarian tissue.

Apoptosis was assessed by the terminal deoxyribonucleotidyl transferase (TdT)-mediated dUTP nick-end labeling (TUNEL) assay, using the tetramethylrhodamine (TMR) red *in situ* Cell Death Detection kit (Roche Diagnostics, Risch-Rotkreuz, Sweden) as we previously described [78]. The number of apoptotic follicles was also recorded when they had signal granulosa cells (more than two cells).

### Ovarian proteomic profile

The proteomic profile of all conditions was performed by sequential window acquisition of all theoretical spectra-mass spectrometry (SWATH-MS) as detailed in Supplementary Table 4. Multiple regression analyses were carried out to identify the differentially expressed proteins (DEPs) associated with each age group. DEPs were represented by hierarchical clustering, and functional analysis was performed using Gene Ontology (GO) through the WGCNA package in R [79].

### Statistical analysis

To determine the statistical differences between groups, we performed the Kruskal-Wallis test, followed by a Mann-Whitney *U*-test for two-by-two comparisons, in GraphPad Prism v.8.12 (GraphPad Software, San Diego, CA, USA). *P* < 0.05 was considered statistically significant. For proteomic analysis, a multiple regression model with ElasticNET penalty was performed using the miXOmics R package (v.6.16.3) [80].

## Supplementary Materials

Supplementary Figure 1

Supplementary Tables
