# Supplementary material for: Deciphering reproductive aging in women using a NOD/SCID mouse model for distinct physiological ovarian phenotypes
Source: Aging (Albany NY). 2023 Oct 16;15(20):10856–74. doi: 10.18632/aging.205086 (PMC10637815; doi:10.18632/aging.205086)
Supplement: Supplementary Figure 1 [file aging-15-205086-s001.pdf]

SUPPLEMENTARY FIGURE

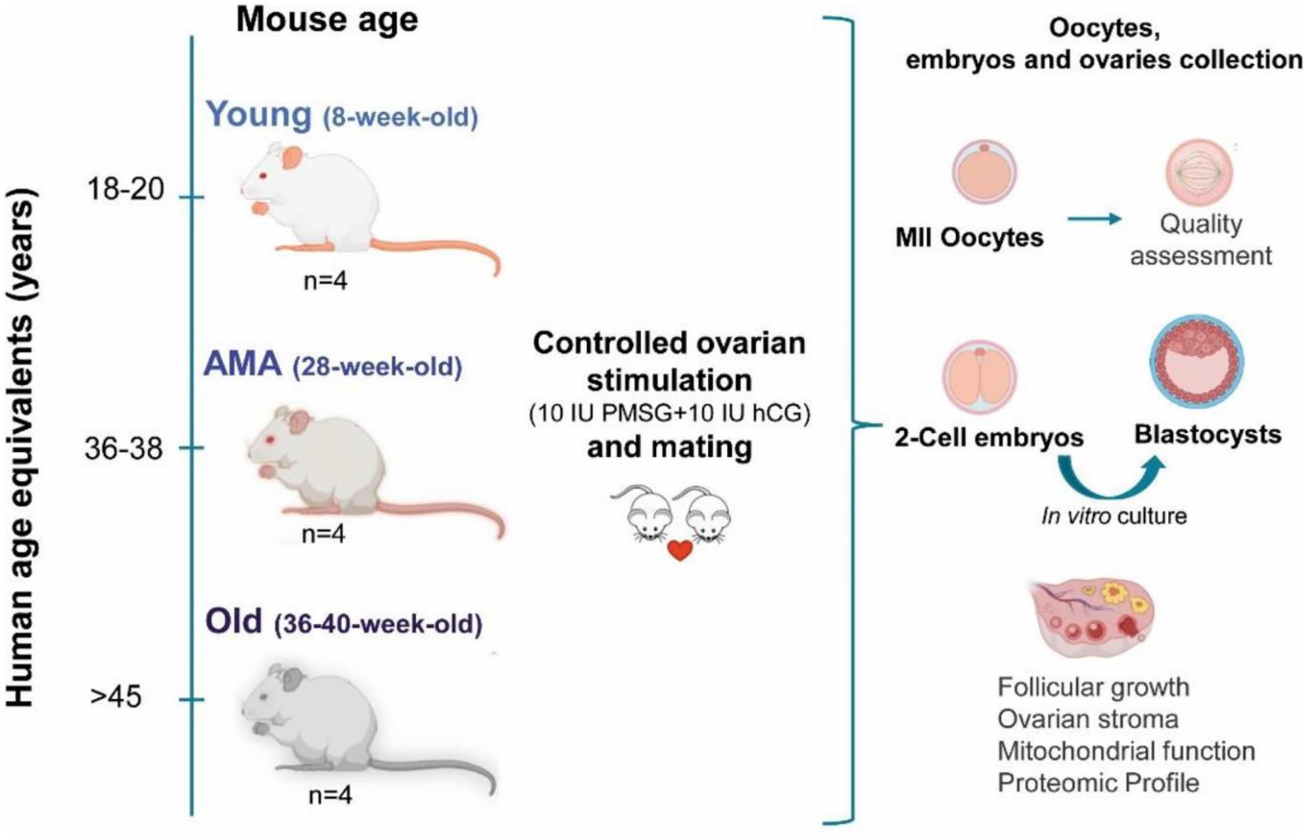

Supplementary Figure 1. NOD/SCID mouse model for physiological human aging. Mice represented distinct reproductive stages of women: young (18-20 years old), Advance Maternal Age (AMA; 36-38 years old), and old (>45 years old). Mice were exogenously stimulated, mated, and sacrificed to recover metaphase-II (MII) oocytes, 2-cell embryos, and ovaries to analyze the short-term effect of age on fertility. Abbreviations: hCG: human chorionic gonadotropin; IU: International Unit; PMSG: pregnant mare serum gonadotropin.
