# Supplementary material for: Deciphering reproductive aging in women using a NOD/SCID mouse model for distinct physiological ovarian phenotypes
Source: Aging (Albany NY). 2023 Oct 16;15(20):10856–74. doi: 10.18632/aging.205086 (PMC10637815; doi:10.18632/aging.205086)
Supplement: Supplementary Tables [file aging-15-205086-s002.pdf]

SUPPLEMENTARY TABLES

Please browse Full Text version to see the data of Supplementary Table 1.

Supplementary Table 1. Proteome composition of the ovarian samples established by SWATH-MS.

Table supplied in excel format: Supplementary Table 1.xlsx.

Supplementary Table 2. Composition of fixing and blocking solutions for oocyte quality assessment.

Solution	Components
	PHEM buffer (60 mM PIPES at pH 6.9, 25 mM HEPES, 10 mM EGTA, 2 mM MgCl2)
	3.7% paraformaldehyde
	50% Deuterium oxide
**Fixing solution***	0.1% Triton X-100
	0.01% aprotinin
	1 mM DTT
	1 µM Taxol
	1X PBS
	1% BSA
	0.2% dried milk
**Blocking solution***	2% Fetal Bovine Serum
	0.1 M Glicina
	0.2% sodium azide
	0.01% Triton x-100

*All reagents from Sigma-Aldrich, St. Louis, MO, USA.

Supplementary Table 3. Primer sequences for the amplification of mitochondrial and nuclear DNA genes and the cycling parameters for RT-qPCR.

Gene	Primer sequences
**ND1**	F:5′-CTAGCAGAAACAAACCGGGC-3′
	R: 5′-CCGGCTGCGTATTCTACGTT-3′
**COX3**	F:5′-TTTGCAGGATTCTTCTGAGC
	R: 5′-TGAGCTCATGTAATTGAAACACC-3′
**18S**	F:5′-CCGCTAGAGGTGAAATTCTT-3′
	R: 5′-CTCCGACTTTCGTTCTTGAT-3′
	**RT-PCR CYCLING PROTOCOL**

50°C for 2 min, dual-lock DNA polymerase for 2min at 95°C, followed by 40 cycles of denaturation for 15 s at 95°C, and annealing and extension for 1min at 60°C.

Supplementary Table 4. SWATH-MS proteomic analysis workflow.

Steps	Procedures details
**Sample preparation**	Lysis: Samples were lysed by the Sample Grinding Kit (GE) with 100 µL of Lysis Buffer (EasyPep™ Mini MS Sample Prep Kit, Thermo Scientific) according to manufacturer's instructions.<br>Protein quantification. After centrifugation (15 minutes at 15,000 g) to remove lipids, 1 µL of every solution was quantified by Qubit protein quantitation kit (Invitrogen) according to manufacturer instructions. Protein concentrations ranged from 4 to 9 µg/µL.<br>Protein digestion: 20 µg of each individual samples were digested using EasyPep™ Mini MS Sample Prep Kit (Thermo Scientific), according to the manufacturer's instructions. After digestion, the peptides were cleaned with the same kit. The peptide mixtures were dried in a speed vacuum and re suspended with 2 % ACN; 0.1% TFA (25 µL) to final theoretical concentration of 0.8 µg/µL.
**SWATH analysis**	LCMSMS-DIA Analysis. For every mixture of digested peptide, 3 µl of peptide mixture sample was loaded by a 425 nanoLC (Eksigent) onto a trap column (3 µ C18-CL, 350 µm x 0.5 mm; Eksigent) and desalted with 0.1% TFA at 5 µl/min during 5 min. Then, the peptides were eluted onto an analytical column (3 µ C18-CL 120 Ă, 0.075 × 150 mm; Eksigent) equilibrated in 5% acetonitrile 0.1% FA (formic acid). Peptide elution was carried out with a linear gradient of 7 to 40% B in 60 min (A: 0.1% FA; B: ACN, 0.1% FA) for at a flow rate of 300 nl/min. Peptides were analysed in a mass spectrometer nanoESI qQTOF (6600plus TripleTOF, ABSCIEX).<br>Sample ionization in a Source Type: Optiflow <1 uL Nano applying 3.0 kV to the spray emitter at 200ºC. The tripleTOF was operated in swath mode, in which a 0.050-s TOF MS scan from 350–1250 m/z was performed. After, 0.080-s product ion scans in 100 variable windows from 400 to 1250 m/z were acquired throughout the experiment. The total cycle time was 2.79 secs.<br>The individual SWATH injections were randomized to avoid bias in the analysis.
**Data analysis protein quantitation**	Wiff files obtained from the SWATH experiment were analyzed by Peak View 2.1 with the user's spectral library according to the following workflow:<br>Target peptide assay (from shotgun identification library).<br>Extract target peptide assay.<br>Score target peptide assay (spectronaut raw score = 3.15).<br>Decoy peptide assay (pseudo-reversed sequence).<br>Extract decoy peptide assay.<br>Score decoy peptide assay (spectronaut score = −1.57).<br>Compare the distribution of targets and decoy scores (FDR estimation for a given score cutoff).<br>Shared and modified peptides were excluded from the analysis.<br>Retention times were aligned among the different samples using main protein peptides. The processing settings used for the peptide selection were:<br>Number of peptides per protein: 20.<br>Number of transitions per peptide: 6.<br>Peptide confidence threshold % (0–99): 95.<br>False discovery rate threshold % (0–100): 1.0.<br>XIC extraction window (min): 20.<br>XIC width (ppm): 50.<br>The cycle time used in the MS-MS/MS acquisition allows quantitate of each peptide area with more than 9 points.<br>The area data obtained with Peak View was analyzed with Marker View (Sciex). First, the calculated protein areas were normalized by the total sum of the areas of all quantified proteins. Next, multiple regression statistical tests were carried out to obtain differentially expressed proteins (DEPs) associated with plasma treatment per age group.

Experimental details of the SWATH-MS technique.
